# TMPRSS4 is a novel biomarker and correlated with immune infiltration in thyroid carcinoma

**DOI:** 10.1186/s12902-022-01203-3

**Published:** 2022-11-16

**Authors:** Xiaoqin Xu, Ting Sun, Jiexian Jing

**Affiliations:** Department of Etiology, Shanxi Province Cancer Hospital, Shanxi Hospital Affiliated to Cancer Hospital, Chinese Academy of Medical Sciences, Cancer Hospital Affiliated to Shanxi Medical University, Taiyuan, Shanxi People’s Republic of China

**Keywords:** Thyroid carcinoma, TMPRSS4, Tumor immune infiltration, Lymphocytes, Prognosis

## Abstract

Transmembrane protease serine 4 (TMPRSS4) is a cancer-associated protease associated with prognosis in various types of cancer. Mechanistically, TMPRSS4 mainly regulates malignant phenotypes, such as tumor invasion and metastasis, by either the epithelial to mesenchymal transition (EMT) program or promoting the proliferation of cancer cells. To date, TMPRSS4 and immune infiltration in thyroid carcinoma (TC) are largely unknown. Thus, this paper evaluated the expression of TMPRSS4 in tumor tissue through the Tumor Immune Estimation Resource (TIMER) database, and Oncomine, and its correlation with clinical parameters by UALCAN databases. Furthermore, we analyzed its prognostic value from Kaplan-Meier Plotter database, and the relationship between TMPRSS4 and the abundance of tumor-infiltrating lymphocytes (TILs) in TC in TISIDB, screening potential immune targets to explore novel mechanisms for the clinical management of TC. Finally, we assessed the correlation between TMPRSS4 and some immune markers to uncover a potential immune-related biomarker in TC patients by TIMER2.0. The results revealed that TMPRSS4 was highly expressed in TC and was also associated with lymphatic metastasis, advanced stage, histological subtype, and favorable clinical outcome. The stratified analysis based on immune cell content showed that decreased TMPRSS4 had worse prognosis in CD8^+^ T cell-enriched TC patients. TMPRSS4 was positively correlated with tumor immune infiltration and the expression of gene markers of immune cells. Notably, its expression was lower in the lymphocyte-depleted subtype than in other immunosubtypes in TC. Moreover, TMPRSS4 was closely related to chemokines as well as their receptors and the immunosuppressive checkpoints CTLA-4, PD-1, and HLA-G. In conclusion, TMPRSS4 may act as a novel biomarker predicting prognosis and immune infiltration in TC.

**Supplementary Information:**

The online version contains supplementary material available at 10.1186/s12902-022-01203-3.

## Introduction

Thyroid carcinoma (TC) is the most common endocrine malignancies. In the past few decades, the incidence of TC cases has sharply increased globally and seriously threatens public health [1]. Although TC is dominated by differentiated thyroid carcinoma (DTC) characterized by a favorable prognosis, radioactive iodine (RAI)-resistant/refractory (RAI-R) TC constitutes a poor-prognosis subgroup within TC, including iodine resistant DTC cases with distant metastasis or recurrence, most anaplastic thyroid carcinoma (ATC) patients and poorly differentiated thyroid cancer (PDTC) [2, 3]. Similarly, approximately 30% of medullary thyroid carcinoma (MTC) cases suffer therapeutic resistance post-surgery. The tumoral intrinsic resistance to therapy, molecular heterogeneity responsible for TC and systematic toxicity caused by drugs limited clinical benefits of targeted therapy. Therefore, it remains essential to explore novel and effective treatment approaches for the management of TC.

The transmembrane protease, serine 4 (TMPRSS4), is a member of the serine protease family and is located in the long arm of chromosome 11 (11q23.3). TMPRSS4 protein harbors proteolytic, stem, transmembrane, and cytoplasmic domains. Abundant evidence revealed that TMPRSS4 played an oncogenic role in various types of cancer, including pancreatic carcinoma [4], breast cancer [5], gastric cancer [6, 7], lung cancer [8], prostate cancer [9], liver cancer [10] colon cancer [11], and TC [12]. Clinically, increased TMPRSS4 expression displayed the potential diagnostic efficiency for pancreatic carcinoma (AUC = 0.91) [4], which was associated with adverse clinical outcome. TMPRSS4 predicted the unfavorable prognosis of stage III-IV colorectal cancer [13] and ESCC [14]. Patients with high expression of TMPRSS4 had shorter overall survival (OS) and disease-free survival (DFS) in breast cancer [15]. Functionally, dysregulated TMPRSS4 mainly controled cell biological behavior and results in tumor proliferation, invasion, and migration phenotype. Elevated TMPRSS4 enhanced the invasive ability of gastric cancer cells through activation of NF-κB and induction of MMP-9 expression [7]. TMPRSS4 modulated both invasion and proliferation via Slug and cyclin D1 [9] Increased TMPRSS4 was responsible for invasion largely through an intracellular signaling cascade that activates FAK, ERK1/2, Akt, Src, and Rac1 in colon cancer [16]. In NCSLC, TMPRSS4 can trigger cancer cell invasion and metastasis via the TMPRSS4/miR-205/ITG-a5 axis [17]. TMPRSS4 accelerated tumor invasion by promoting uPA gene expression or transferring pro-uPA into its active form in lung and prostate cancer [18].TMPRSS4 can promote HCC proliferation, invasion, and angiogenesis by regulating HB-EGF [19].

In TC, upregulated TMPRSS4 accelerated cancer cell proliferation by mediating CREB phosphorylation [12]. More importantly, TMPRSS4 has excellent performance for the diagnosis of TC. Kebebew E et al. [20] demonstrated that upregulated TMPRSS4 was a potential powerful marker for the diagnosis of malignant thyroid nodules (AUC = 0.926), which combined with ECM1 might improve the diagnostic accuracy of FNA biopsy (AUC = 0.985). Similarly, Zhang Y et al. found that TMPRSS4 had good diagnostic performance of TMPRSS4 for TC(sensitivity: 93.33%, specificity: 100%, accuracy: 96.7%) , which combined with BRAF mutation improved the diagnostic sensitivity to 97.62% and the accuracy to 97.73% [21]. In principle, the active TMPRSS4 protease can be secreted and released into the cell culture medium, and it may be detected in human serum samples. Hence, TMPRSS4 may serve as a promising mininvasive diagnostic marker. Moreover, TMPRSS4 was reported to be involved in viral infection [22], indicating that it is likely to be associated with host immune regulation. Autoimmune thyroid disorders and TC can be concurrent. Accumulating evidence showed that several immune disorders are involved in this transforming and evolving progress of TC [23]. However, its role and mechanism for regulating the tumor microenvironment remain inclusive.

In our present study, we performed a comprehensive assessment of the expression profiles and clinical significance of TMPRSS4 by analysis from public databases such as Oncomine, TIMER, and the Kaplan–Meier plotter, UALCAN. Finally, we explored the relationship between TMPRSS4 and tumor-infiltrated immune cells using TIMER and TISIDB. We also analyzed its correlation with chemokines, their receptors, and some immune inhibitors. Our results not only offer new insights into the function of TMPRSS4 in TC but also highlight a possible molecular mechanism of the interaction between TMPRSS4, immune cells and tumor cells. The best understanding of TMPRSS4 will provide some clues for favoring immunotherapy for TC.

## Materials and methods

### Oncomine database analysis

The Oncomine database is a single comprehensive database integrating 86,733 samples, 715 gene expression datasets, and 19 cancer types. It’s designed to screen differentiated expressed genes, co-expression genes by data mining. We analyzed the expression profile of TMPRSS4 in tumors by using this database (https://www.oncomine.org/resource/main.html).

### TIMER database analysis

TIMER2.0 web server (http://timer.cistrome.org/) is a comprehensive resource for systematical analysis of immune infiltrates across diverse cancer types. The abundances of six immune infiltrates (B cells, CD4^+^ T cells, CD8^+^ T cells, Neutrophils, Macrophages, and Dendritic cells (DCs)) are estimated by TIMER algorithm. We explore the differential expression between tumor and adjacent normal tissues for any gene of interest across all TCGA tumors by Gene_DE module, the correlation between TMPRSS4 expression and the abundance of immune infiltrates based on the different methods for immune-oncology (TIMER, xCell, CIBORSORT, etc.) in the gene module, the association between clinical outcome and abundance of immune infiltrates or gene expression in the Outcome module, and the correlations between TMPRSS4 expression and some particular markers associated with immune cell infiltration of tumors in the Gene_Corr module.

### Kaplan–Meier plotter analysis

Kaplan–Meier plotter consists of GEO, EGA, and TCGA database (http://kmplot.com/analysis), which serves as an analysis tool for assessing the effect of 54 k genes (mRNA, miRNA, and protein) on survival in 21 cancer types based on discovery and validation of survival biomarkers. RNA sequencing (RNA-seq) data were utilized from the Cancer Genome Atlas (TCGA, https://cancergenome.nih.gov/). We explore the predicting role of TMPRSS4 expression for prognosis in multiple types of cancer patients and specific immune cell-enriched TC patients by employing the pan-cancer Kaplan–Meier plotter database with the best performing cutoff value.

### TISIDB database analysis

TISIDB is a web database integrating diverse heterogeneous data types to analyze the interaction between tumor and the immune system (http://cis.hku.hk/TISIDB/index.php). We conduct the correlation analysis between TMPRSS4 and the abundance of 28 tumor-infiltrating lymphocytes (TILs) in TC. And its correlation with various chemokines and their related receptor, and immunoinhibitors.

### UCLCAN database analysis

UALCAN is a web portal containing abundant cancer OMICS data (http://ualcan.path.uab.edu/index.html). We perform the further validation of the analysis in the former database, compare the expression of TMPRSS4 in TC with its corresponding normal tissue, and confirm the correlation of TMPRSS4 expression and the clinical features.

### Statistical analysis

We performed the corresponding analysis in above-mentioned database. Spearman's correlation analyses were used to explore the correlation between particular variables, with r values for judging the strength of correlation. *P* < 0.05 was determined as statistical significance.

## Results

### TMPRSS4 was highly expressed in TC tissue

**Fig. 1:**
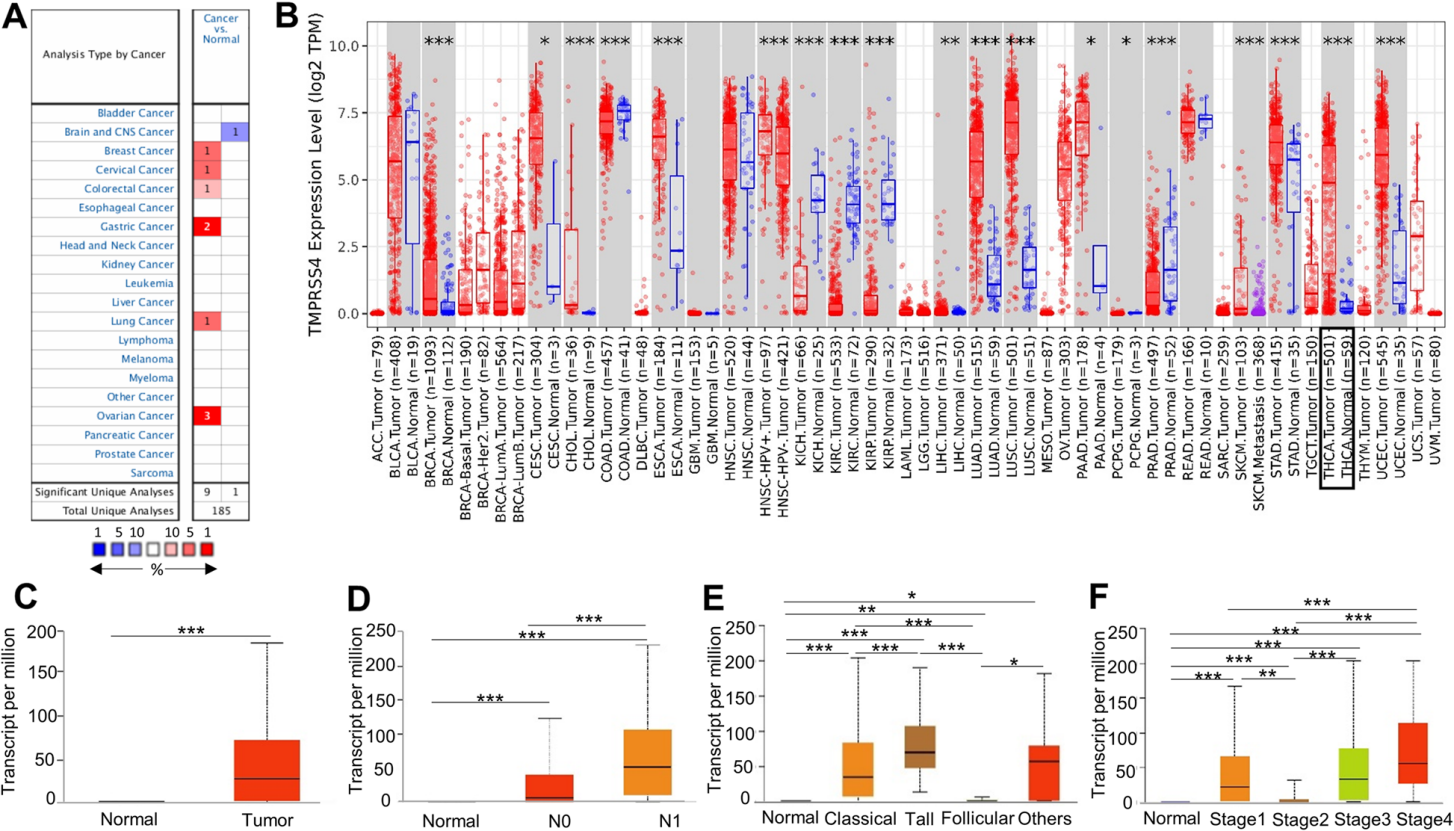
Aberrant expression of TMPRSS4 and correlation with malignant features of TC patients in TCGA. **A** and **B** The relative expression level of TMPRSS4 mRNA in cancer from Oncomine and TIMER2.0. **C** Comparison of TMPRSS4 expression level between TC tissue (*n* = 505) and normal control (*n* = 59) from TCGA database by UALCAN. **D** Comparison of TMPRSS4 expression level in different lymph node metastasis status (Normal: *n* = 59, N0: *n *= 230, N1: *n* = 58) in TC patients from TCGA database by UALCAN. **E** Comparison of TMPRSS4 expression level in different histological subtype group including classical PTC (*n* = 358), tall cell PTC (*n* = 36), follicular PTC (*n *= 102), and others (*n* = 9) in TC patients from TCGA database by UALCAN. **F** Comparison of TMPRSS4 expression level in different tumor stage (Normal: *n* = 59, Stage1/2/3/4: *n* = 284/52/112/55) in TC patients collected in TCGA database by UALCAN. *: *P* < 0.05, **: *P* < 0.01, and ***: *P* < 0.001. ACC, adrenocortical carcinoma; BLCA, bladder urothelial carcinoma; BRCA, breast invasive carcinoma; CESC, cervical and endocervical cancer; CHOL, cholangiocarcinoma; COAD, colon adenocarcinoma; ESCA, esophageal carcinoma; GBM, glioblastoma multiforme; HNSC, head and neck squamous cell carcinoma; KICH, kidney chromophobe; KIRC, kidney renal clear cell carcinoma; KIRP, kidney renal papillary cell carcinoma; LAML, acute myeloid leukemia; LGG, lower grade glioma; LIHC, liver hepatocellular carcinoma; LUAD, lung adenocarcinoma; LUSC, lung squamous cell carcinoma; MESO, mesothelioma; OV, ovarian serous cystadenocarcinoma; PAAD, pancreatic ductal adenocarcinoma; PCPG, paraganglioma and pheochromocytoma; PRAD, prostate adenocarcinoma; READ, rectum adenocarcinoma; SARC, sarcoma; SKCM, skin cutaneous melanoma; STAD, stomach adenocarcinoma; TGCT, testicular germ cell tumors; THCA, thyroid carcinoma; THYM, thymoma; UCEC, uterine corpus endometrial carcinoma; UCS, uterine carsinosarcoma; UVM, uveal melanoma

We assessed the mRNA expression level of TMPRSS4 across cancers using two independent databases, the Oncomine and TIMER databases, and found that the expression levels of TMPRSS4 differed in various cancers (Fig. 1A and B). TMPRSS4 was increased in most cancers from the Oncomine database compared with normal tissue (Fig. 1A). Notably, it was elevated in BRCA, CESC, CHOL, ESCA, LIHC, LUAD, LUSC, PAAD, STAD, THCA, and UCEC, while there was decreased TMPRSS4 in COAD, KICH, KIRH, KIRC, PGPC, and PRAD (Fig. 1B). This finding is indicative of the different biological roles of TMPRSS4 in various types of cancer. Specifically, TMPRSS4 was remarkably upregulated in TC tissue compared with normal tissue (Fig. 1B), which was validated by the UALCAN database (Fig. 1C). All these results indicated that the expression of TMPRSS4 in TC was in line with previous research. In addition, TMPRSS4 expression was higher in TC patients having lymph node involvement than normal controls (Fig. 1D). It was associated with the histological type (Fig. 1E) and advanced stage of TC from TCGA database (Fig. 1F). Notably, our study evaluated only carcinomas which originate from the thyrocytes, and TMPRSS4 was more highly expressed in tall cell variant of papillary thyroid carcinoma (PTC) than in classical and follicular PTC. This finding was indicative of the relationship between TMPRSS4 expression and malignant features.

### The predicting role of TMPRSS4 for prognosis of TC

**Fig. 2:**
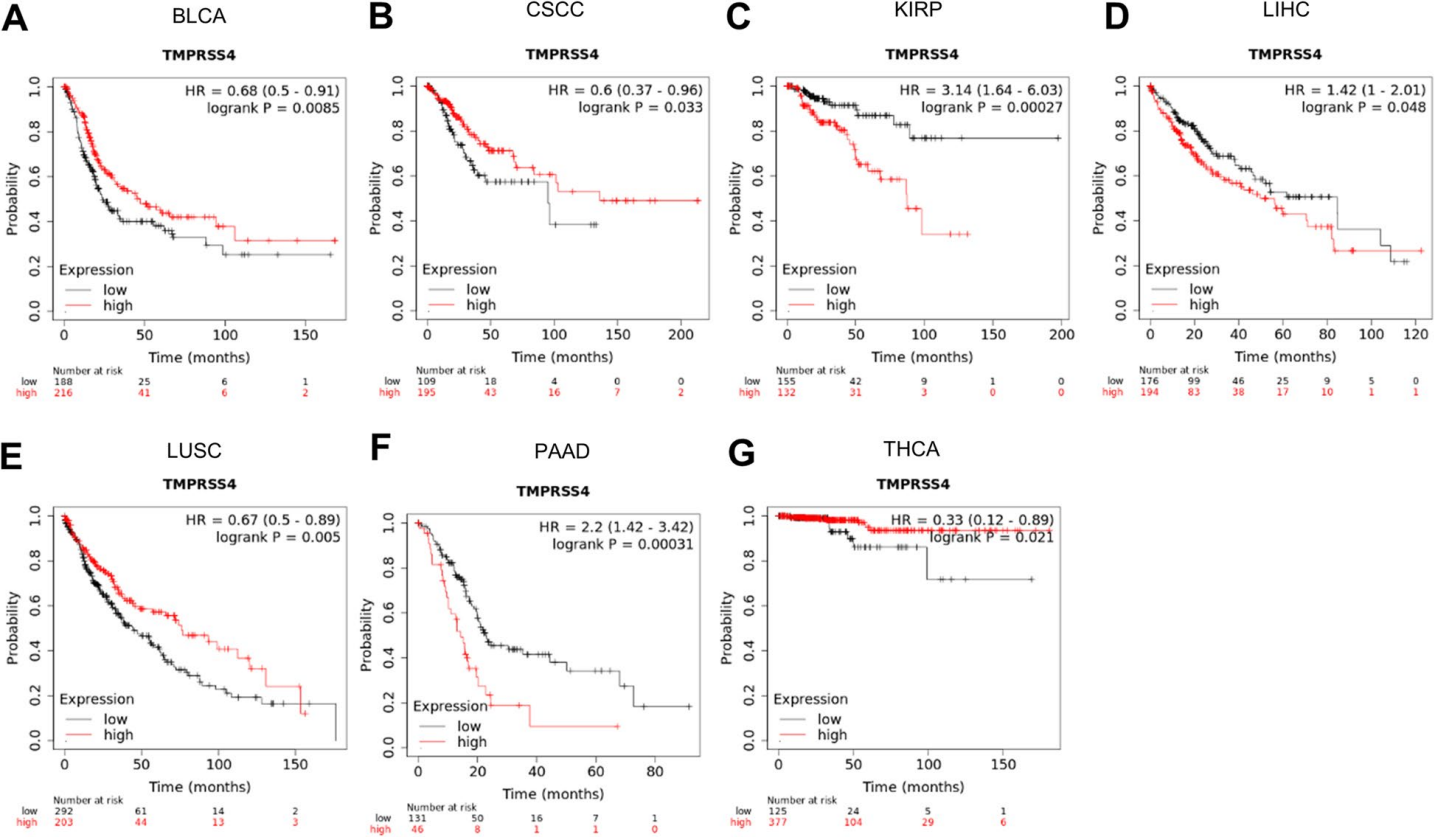
The survival analysis of high and low expression of TMPRSS4 for various types of tumor by Kaplan–Meier Plotter. **A**, **B**, **C**, **D**, **E**, **F**, and **G** presented the prognostic role of TMPRSS4 for BLCA, CSCC, KIRP, LIHC, LUSC, PAAD, THCA based on TCGA database by Kaplan–Meier Plotter, respectively

**Fig. 3:**
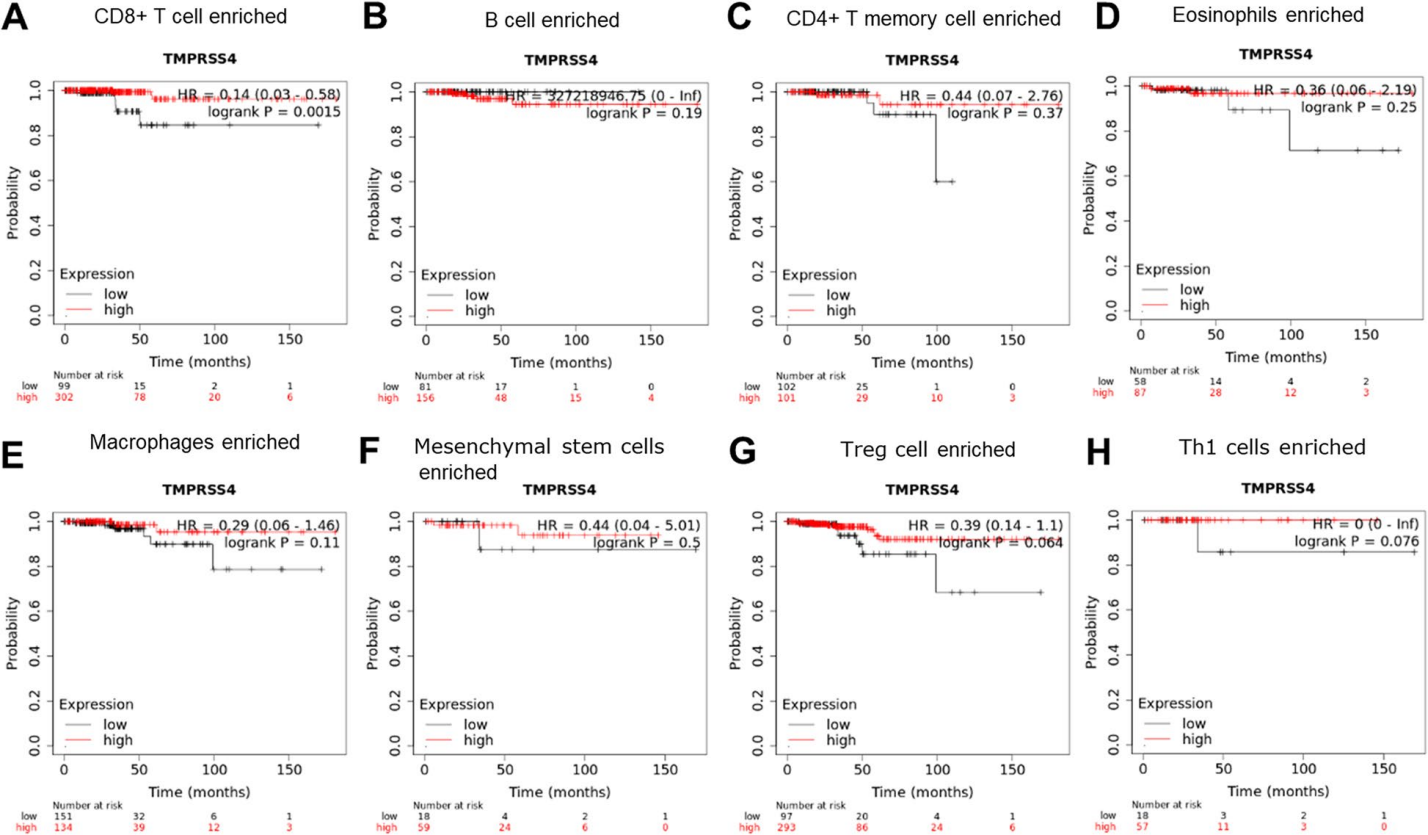
The survival analysis of TMPRSS4 expression after stratified by immune cell content using Kaplan–Meier Plotter. **A**, **B**, **C**, **D**, **E**, **F**, and **G** presented the prognostic role of TMPRSS4 when conditioned on CD8^+^ T cell-enriched, B cell-enriched, CD4^+^ T memory cell-enriched, eosinophils-enriched, macrophage-enriched, mesenchymal stem cells-enriched, Treg cell-enriched, Th1 cell-enriched THCA based on TCGA database by Kaplan–Meier Plotter, respectively

We employed the pan-cancer Kaplan–Meier plotter database and analyzed the association between TMPRSS4 expression and clinical outcome. We found that the predictive role of TMPRSS4 for prognosis varied in different types of cancers. Among them, decreased TMPRSS4 predicted a poor prognosis for BLCA, cervical squamous cell carcinoma (CSCC), LUSC, and THCA. In contrast, patients with elevated TMPRSS4 in KIRP, LIHC, and PAAD had a shorter OS time (Fig. 2). Given the complex tumor microenvironment (TME), we further investigated the prognostic predictive role of TMPRSS4 in different lymphocyte-enriched THCA patients (Fig. 3). The results showed that THPA patients with decreased TMPRSS4 had worse clinical outcomes when CD8^+^ T-cells were enriched. However, there was no remarkable statistical discrepancy regarding OS between high and low TMPRSS4 expression group when other immune cells were enriched in THCA, such as B cells, CD4^+^ T cells, eosinophils, macrophages, mesenchymal stem cells, regulatory T cells (Treg), and type 1 T-helper cells (Th1). Unfortunately, there was a limitation that clinical samples of enriched type 2 T-helper cells (Th2) cells and natural killer T (NKT) cells were too small to analyze further.

### The correlation between TMPRSS4 and the abundance of TILs

**Fig. 4:**
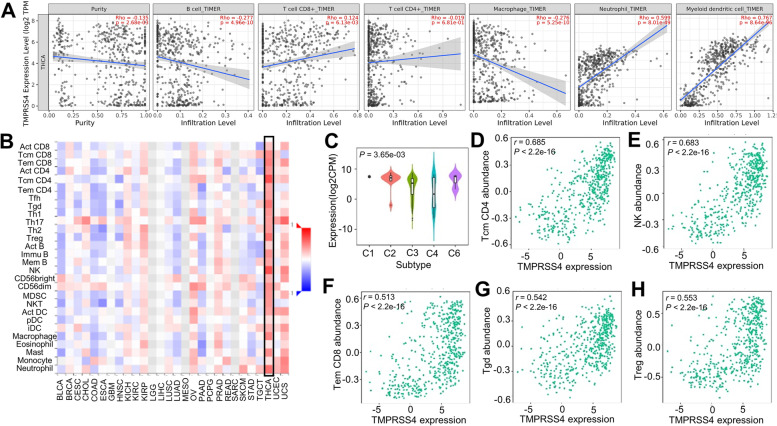
The association between TMPRSS4 and tumor immune infiltration in TC. **A** The correlation analysis between TMPRSS4 and six TILs from TIMER2.0. **B** Analysis of correlation between expression of TMPRSS4 and abundance of TILs in various types of human cancers from TISIDB, X axis represented various types of human cancer, Y axis represented TILs, the color of the cell in the heatmap represented the degree of their correlation based on the rho value. **C** The expression of TMPRSS4 in six immune subtype of TC from TISIDB database. **D**-**H** TMPRSS4 strongly and positively correlated with CD4 Tcm (**D**), NK cells (**E**), Tem CD8 cells (**F**), Tgd (**G**), and Treg (**H**) in TC

TC is associated with immune dysregulation of the thyroid microenvironment. We explored the cross-talk between TMPRSS4 and immune infiltration in the TC microenvironment and indicated that TMPRSS4 showed a positive correlation with most immune cells, such as CD8^+^ T cells (*r* = 0.124, *P* < 0.01), neutrophils (*r* = 0.599, *P* < 0.001) and DCs (*r* = 0.767, *P* < 0.001), and a weakly negative correlation with B cell (*r* = -0.277, *P* < 0.001) and macrophages (*r* = -0.276, *P* < 0.001) in TC (Fig. 4A, Supplementary Table 1) based on the TIMER2.0 database.

Meanwhile, we determined the correlation between TMPRSS4 and the abundance of TILs by the TISIDB database (Fig. 4B, Supplementary Table 2). The analysis revealed that TMPRSS4 strongly and positively correlated with central memory CD4 T cells (Tcm CD4) (*r* = 0.685, *P* < 0.001) and NK cells (r = 0.683, *P* < 0.001) (Fig. 4 D and E) and moderately associated with effector memory CD8 T cells (Tem CD8) (*r* = 0.513, *P* < 0.001), gamma delta T cell (Tgd) (*r* = 0.542, *P* < 0.001), and Treg (*r* = 0.553, *P* < 0.001) (Fig. 4 F, G, and H). In addition, there was a positive association between TMPRSS4 expression and activated B cells (Act B) (*r* = 0.188, *P* < 0.001), activated CD4 T cells (Act CD4) (*r* = 0.424, *P* < 0.001), activated CD8 T cells (Act CD8) (*r* = 0.247, *P* < 0.001), CD56dim NK cells (CD56 dim) (*r* = 0.442, *P* < 0.001), CD56 bright (CD56 bright) cells (*r* = 0.307, *P* < 0.001), immature B cells (imm B) (*r* = 0.434, *P* < 2.2e-16), memory B cells (mem B) (*r* = 0.356, *P* < 0.001), effector memory CD4 T cells (Tem CD4) (*r* = 0.349, *P* < 0.001), central memory CD8 T cells (Tcm CD8) (*r* = 0.465, *P* < 0.001), T follicular helper cell (Tfh) (*r* = 0.381, *P* < 0.001), Th1 (*r* = 0.417, *P* < 0.001), type 17 T-helper cells (Th17) (*r* = 0.384, *P* < 0.001) and other immune cells (Supplementary Fig. 1). And we also used different methodologies for immune-oncology such as xCell and CIBERSOR for further validation (Supplementary Table 3 and 4). We found the similar positive corelation with Tregs and DC. Based on tumor-infiltrated immune cells, we further explored the distribution of TMPRSS4 expression across immune subtypes and revealed that there was a significantly decreased TMPRSS4 in the lymphocyte-depleted subtype when compared to the other subtypes (wound healing, IFN-gamma dominant, inflammatory, immunologically quiet, TGF-β dominant) (Fig. 4C). These analyses indicated that there may be a fine-tuned regulatory role between TMPRSS4 expression and TILs in the carcinogenesis and development of TC.

### TMPRSS4 correlated with the gene marker of immune cells

We further evaluated the correlation of TMPRSS4 expression with the markers of each tumor infiltrating immune cells in TC from the TIMER2.0 database. The analysis illustrated that TMPRSS4 was associated with most of the immune cells (Table 1), including B cells, CD8^+^ T cells, DCs, M1 macrophages, M2 macrophages, monocytes, neutrophils, T cells (general), T cell exhaustion, TAMs, Tfhs, Th1 cells, Th2 cells, Th17 cells, and Tregs. Meanwhile, considering that tumor purity is a major confounding factor in this analysis, we adjusted the analysis results by tumor purity and pinpointed out a significant correlation between TMPRSS4 expression and B cell markers (CD19 and CD79A), CD8^+^ T cells (CD8A and CD8B), DCs markers (ITGAX, CD1C, HLA-DPA1, HLA-DRA, HLA-DQB1, and HLA-DPB1), M1 macrophages markers (PTGS2 and IRF5), M2 macrophages markers (CD163, VSIG4, and MS4A4A), monocyte markers (CSF1R and CD86), neutrophils markers (CCR7, ITGAM, and CEACAM8), T cell general markers (CD3D, CD3E, and CD2), T cell exhaustion markers (CTLA4, LAG3, HAVCR2, GZMB, and PDCD1), TAMs markers (CCL2, IL10, CD68, LAG3, HAVCR2, and GZMB), the Tfh marker BCL6, Th1 markers (TBX21, STAT4, STAT1, IFNG, and IL13), Th2 markers (GATA3, STAT6, and STAT5A), Th17 markers (STAT3 and IL17A), and Treg markers (FOXP3, CCR8, and TGFB1). Undoubtedly, all these analysis results revealed that TMPRSS4 might be related to the onset and progression partially through regulating immune infiltration.

**Table 1:** The correlation between TMPRSS4 and immune cells-related gene markers in THCA from the TIMER2.0 database

**Immune cells**	**Markers**	**None**		**Purity**	
		***r***	***P***	***r***	***P***
B cell	CD19	0.219	6.05E-07***	0.204	5.44E-06***
	CD79A	0.289	2.85E-11***	0.275	6.34E-10***
CD8^+^ T cell	CD8A	0.099	2.49E-02*	0.096	3.38E-02*
	CD8B	0.327	3.81E-14***	0.335	3.14E-14***
Dendritic cell	ITGAX	0.468	4.50E-29***	0.453	4.33E-26***
	NRP1	0.007	0.8825	-0.003	0.945
	CD1C	0.585	3.85E-48***	0.567	7.43E-43***
	HLA-DPA1	0.428	4.54E-24***	0.411	2.46E-21***
	HLA-DRA	0.443	7.18E-26***	0.424	1.01E-22***
	HLA-DQB1	0.378	9.13E-19***	0.37	2.59E-17***
	HLA-DPB1	0.395	1.71E-20***	0.38	3.65E-18***
M1 Macrophage	PTGS2	0.665	3.28E-66***	0.663	4.21E-63***
	IRF5	0.590	4.52E-49***	0.586	2.99E-46***
	NOS2	0.034	0.4385	0.041	0.372
M2 Macrophage	MS4A4A	0.400	5.15E-21***	0.385	1.12E-18***
	VSIG4	0.399	7.22E-21***	0.388	5.68E-19***
	CD163	0.382	4.39E-19***	0.368	4.59E-17***
Monocyte	CSF1R	0.299	5.61E-12***	0.287	9.83E-11***
	CD86	0.438	3.20E-25***	0.421	2.03E-22***
Natural killer cell	KIR2DS4	-0.096	3.02E-02*	-0.094	3.72E-02*
	KIR3DL3	0.009	0.844	0.011	0.815
	KIR3DL2	0.018	0.684	0.006	0.903
	KIR3DL1	-0.098	0.027*	-0.101	2.57E-02*
	KIR2DL4	-0.107	0.016*	-0.112	0.013*
	KIR2DL3	-0.033	0.462	-0.037	0.416
	KIR2DL1	-0.156	4.01E-04***	-0.144	1.44E-03**
Neutrophils	CCR7	0.404	2.07E-21***	0.384	1.38E-18***
	ITGAM	0.496	6.84E-33***	0.48	1.7E-29***
	CEACAM8	0.408	7.23E-22***	0.405	1.23E-20***
T cell(general)	CD3D	0.344	1.33E-15***	0.334	3.24E-14***
	CD3E	0.355	1.49E-16***	0.344	5.21E-15***
	CD2	0.381	4.53E-19***	0.367	5.72E-17***
T cell exhaustion	CTLA4	0.459	7.40E-28***	0.445	4.7E-25***
	LAG3	0.253	6.94E-09***	0.252	1.58E-08***
	HAVCR2	0.424	1.19E-23***	0.408	5.25E-21***
	GZMB	0.151	6.39E-04***	0.149	9.6E-04***
	PDCD1	0.074	0.093	0.083	6.85E-02
TAM	CCL2	0.336	6.91E-15***	0.322	2.9E-13***
	IL10	0.307	1.55E-12***	0.284	1.69E-10***
	CD68	0.484	3.13E-31***	0.465	1.42E-27***
Tfh	BCL6	0.350	4.31E-16***	0.333	4.01E-14***
	IL21	0.056	0.205	0.058	0.2
Th1	TBX21	0.111	0.012*	0.104	2.19E-02*
	STAT4	0.490	4.01E-32***	0.486	3.11E-30***
	STAT1	0.578	1.14E-46***	0.563	3.94E-42***
	IFNG	0.210	1.72E-06***	0.201	7.77E-06***
	IL13	0.145	1.07E-03***	0.143	1.57E-03**
Th2	GATA3	0.450	8.55E-27***	0.445	4.69E-25***
	STAT6	0.350	4.35E-16***	0.338	1.81E-14***
	STAT5A	0.374	2.18E-18***	0.375	8.75E-18***
Th17	STAT3	0.353	2.41E-16***	0.336	2.4E-14***
	IL17A	0.185	2.70E-05***	0.177	8.36E-05***
Treg	FOXP3	0.537	2.07E-39***	0.521	2.6E-35***
	CCR8	0.488	8.16E-32***	0.472	1.8E-28***
	STAT5B	0.058	0.189	0.055	0.227
	TGFB1	0.316	2.92E-13***	0.316	9.64E-13***

None: the correlation analysis without any adjustment, Purity: the adjusted correlation analysis by tumor purity. *P < 0.05, **P < 0.01, ***P < 0.001

### The relationship between TMPRSS4, chemokines and their receptors

**Fig. 5:**
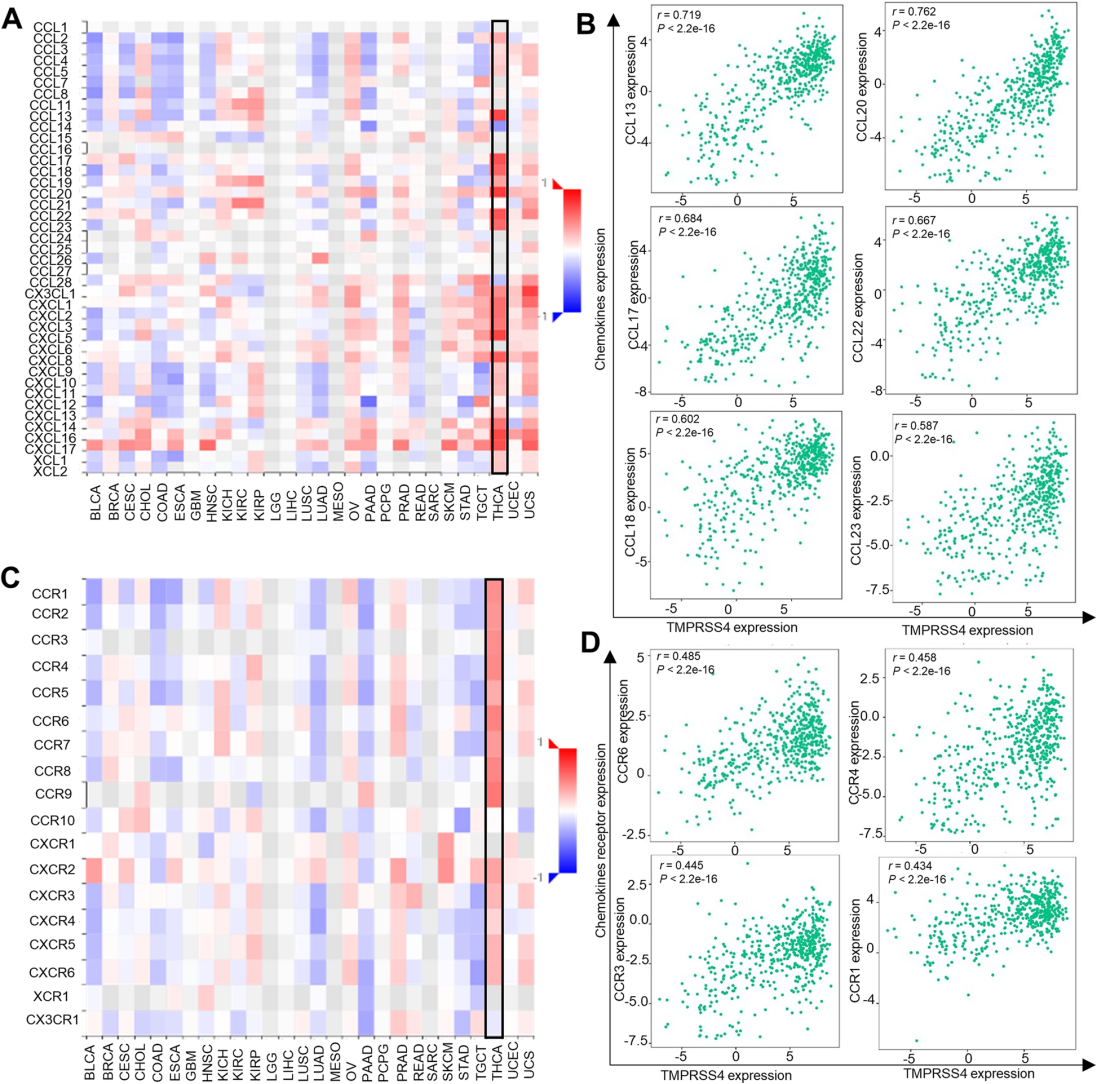
The relationship between TMPRSS4 and chemokines and their receptors in TC from TISIDB. **A** The correlations between TMPRSS4 expression and chemokines across human cancers including TC from TISIDB. X axis represented various types of human cancer, Y axis represented chemokines, the color of the cell in the heatmap represented the degree of their correlation based on the rho value. **B** TMPRSS4 expression was strongly and positively correlated with chemokines CCL13, CCL17, CCL18, CCL20, CCL22, CCL23. **C** The correlations between TMPRSS4 expression and receptors of chemokines across human cancers including TC from TISIDB database. X axis represented various types of human cancer, Y axis represented chemokines receptors, the color of the cell in the heatmap represented the degree of their correlation based on the rho value. **D** TMPRSS4 expression was positively correlated with chemokines receptors CCR6, CCR4, CCR3, CCR1

In addition to abundant immune cells, chemokines also act as a guide for stimulating the recruitment of leukocytes. Previous studies have shown that some chemokines and their related receptors played a significant role in cancer. In TC, highly expressed CCL17, CCL22, and CCL23 protein levels predicted unfavorable outcomes, whereas increased CCL15, CCL8, and CCR2 predicted better prognosis [24]. We further analyzed its correlation with chemokines and corresponding receptors (Fig. 5A, B). The analysis revealed that there was a significant positive correlation between TMPRSS4 and chemokines including CCL13, CCL17, CCL18, CCL19, CCL20, CCL22, CCL23, CXCL1, CXCL2, CXCL3, CXCL5, and CX3CL1, while TMPRSS4 negatively correlated with CCL14 and CCL28 (Fig. 5A, C and Supplementary Fig. 2). Specifically, TMPRSS4 expression strongly correlated with CCL13 (*r* = 0.719, *P* < 0.001), CCL20 (*r* = 0.762, *P* < 0.001), CCL17 (*r* = 0.684, *P* < 0.001), CCL22 (*r* = 0.667, *P* < 0.001), CCL18 (*r* = 0.602, *P* < 0.001) and CCL23 (*r* = 0.587, *P* < 0.001) (Fig. 5C). Moreover, TMPRSS4 positively correlated with CCR1-8 and CXCR2-6 and reversely correlated with CX3CR1 (Fig. 5D and Supplementary Fig. 3). Therefore, we speculated that TMPRSS4 might mediate malignant proliferation and metastasis of TC by modulating chemokine/chemokine receptor axes.

### The relationship between TMPRSS4 and expression of immunosuppressive markers

**Fig. 6:**
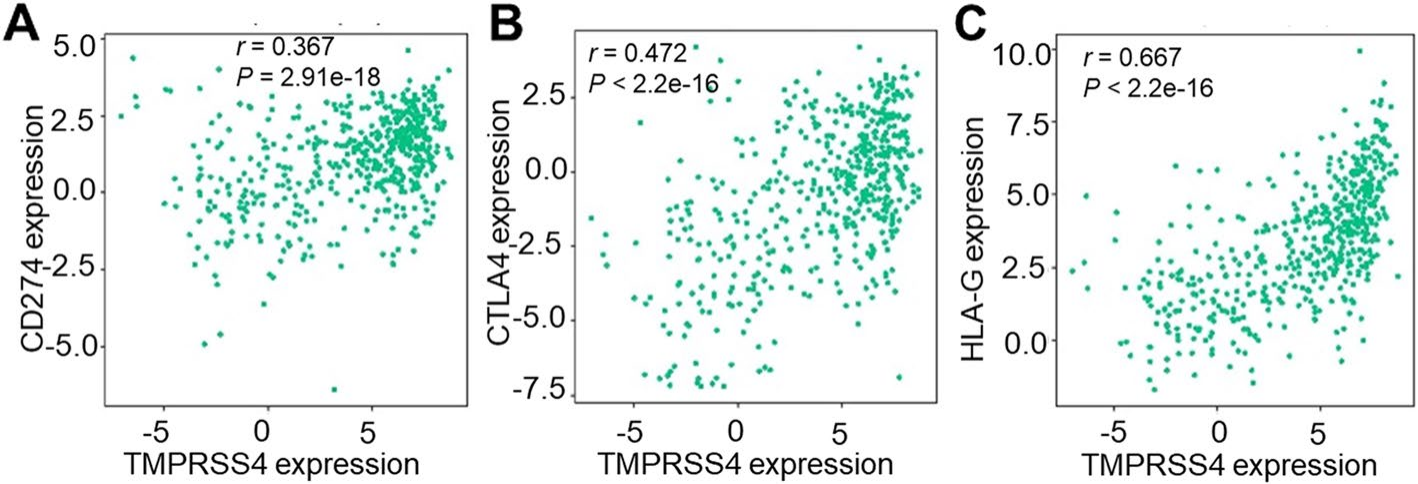
The correlation diagram between TMPRSS4 and expression of immunosuppressive markers. **A**, **B** and **C** represented TMPRSS4 expression positively correlated with the expression of CD274, CTLA-4, and HLA-G, respectively

Immunotherapy has revolutionized the treatment of TC. We analyzed the classical immune inhibitory markers cytotoxic T lymphocyte-associated antigen-4 (CTLA-4), Programmed cell death 1 ligand 1 (PD-L1), and HLA-G. TMPRSS4 displayed positive correlation with CTLA-4 (*r* = 0.472, *P* < 0.001), PD-L1 (*r* = 0.367, *P* < 0.001) and HLA-G (*r* = 0.667, *P* < 0.001)(Fig. 6). This finding was consistent with the correlation analysis in TIMER 2.0 (Supplementary Fig. 4), suggesting that TMPRSS4 will be a potential immunotherapy target.

## Discussion

In this study, we for the first time analyzed the expression level of TMPRSS4 and investigated its correlation with clinical pathological characteristics and tumor immune cell infiltration to assess its prognostic value in TC. Recent studies showed that TMPRSS4 is highly expressed in cancer tissues, and its dysregulation has been implicated in tumorigenesis and malignant progress [4–12]. We performed the analysis in Oncomine databases and found that TMPRSS4 was significantly upregulated in breast cancer, cervical cancer, colorectal cancer, gastric cancer, and ovarian cancer but downregulated in brain and CNS cancer. Similarly, the TIMER2.0 database displayed TMPRSS4 level was elevated in BRCA, CESC, CHOL, ESCA, LIHC, LUAD, LUSC, PAAD, STAD, THCA, and UCEC, while there was decreased TMPRSS4 in COAD, KICH, KIRH, KIRC, PGPC, and PRAD. All these results supported previous research outcomes [4–12]. Altered TMPRSS4 expression across human cancers may result from different data collection manner in diverse studies or distinct underlying biological mechanisms. Meanwhile, the increased TMPRSS4 was highest in tall cell PTC, significantly associated with lymphatic metastasis, higher stage, and tall cell variant of PTC. These results shed light on an essential role in the formation and metastasis of TC. Our findings was consistent with previous studies [12].

Based on the TCGA data, the Kaplan-Meier plotter database revealed upregulated TMPRSS4 levels were correlated with a worse outcome for patients with KIRP, LIHC, and PAAD. In contrary, increased TMPRSS4 positively correlated with the OS of BLCA, CSCC, LUSC. Notably, the survival analysis was in favour of the previous reports in LIHC [25], PADD [4]. Surprisingly, the prognostic role of TMPRSS4 in LUSC by pan-cancer Kaplan-Meier plotter was opposite to the literature [17, 26], which was attributed to data collection method of different dataset, complex clinical context, diverse tumor environment or altered underlying molecular mechanism. Unexpectedly, decreased TMPRSS4 predicted poor prognosis in TC patients from the Kaplan-Meier Plotter analysis. Immune disorders including autoimmune disease increases risk for cancer development [23]. The TME showed complexity and diversity, not only in components but also in specific functions, including abundant immune cells and tumor cells, chemokines and their corresponding receptors, etc [27]. Furthermore, we discovered TMPRSS4 correlated with classical tumor immune cell infiltration (CD4^+^ T cells, B cells, macrophages, DCs, neutrophils) in TC patients from the TIMER2.0 database. TMPRSS4 expression showed strongly positive association with DCs and neutrophils, weakly positive correlation with CD8^+^ T cells, and negatively correlated with tumor purity, B cells, macrophages,. Furthermore, the survival analysis showed that TMPRSS4 may be as a prognostic predictor and protective factor for TC by Kaplan-Meier Plotter. Its prognostic role was arguably attributed to the immune cell infiltration in the TME. We also investigated its predictive value in a lymphocyte enriched cohort of TC and uncovered a similar phenomenon only in CD8^+^ T cell-enriched group by Kaplan-Meier Plotter analysis. Meanwhile, TMPRSS4 expression positively correlated with the abundance of NK cells, Tcm_CD4, Tem_CD8, Tgd, and Treg from the TISIDB database. TMPRSS4 might be involved in antitumor immunity and thus result in a favorable prognosis. In general, the immune subtype was classified into six subtypes: wound healing, IFN-gamma dominant, inflammatory, lymphocyte depleted, immunologically quiet, and TGF-β dominant. Notably, lower TMPRSS4 mRNA expression was shown in the lymphocyte-depleted subtype. It suggested that there was an intrinsic link between TMPRSS4 and tumor immune infiltration. Therefore, we speculated that TMPRSS4 might play an immune-regulatory role in TC.

On the other hand, we performed a similar analysis between TMPRSS4 expression and gene markers of each type of immune cells. TMPRSS4 displayed a significant and positive correlation with most of the markers, except for NK cells. TMPRSS4 may be involved in the progression of TC by regulating innate and adaptive immunity. To date, increasing studies have shown evidence that T cell exhaustion leads to immune escape [28]. Significantly and interestingly, TMPRSS4 was positively correlated with markers of T cell exhaustion. Accordingly, it provides a clue that TMPRSS4 acts as a multifaceted factor, which drives TC progression because of escaping from immune evasion, thereby inducing T cell exhaustion and improving survival for the effective therapeutic response to immune checkpoint inhibitor immunotherapy. It also correlated with markers of T cell subpopulations such as Th1, Th2, Th17, and Tfh in TC, indicating the underlying mechanism between TMPRSS4 expression and the function of T cells in TC. Obviously, there were relatively weak correlations between TMPRSS4 expression and some parameters such as activated CD8 T cell, activated B cell, plasmacytoid DC, eosinophil, and monocyte from TISIDB database, gene marker of B cell and NK cell from TIMER 2.0. It’s implicit to pay more attention to these link for further validation in clinical investigation and exploring its regulatory mechanism in experimental study.

Given the diversity and complexity within the TME, innate immune cells (macrophages, mast cells, neutrophils) and adaptive immune cells (lymphocytes) communicate with cancer-associated cells such as fibroblasts, adipocytes, endothelial cells, and extracellular matrix via chemokines, adipocytokines, and cytokines [24, 29]. Moreover, TMPRSS4 serves as a soluble fragment, which is thereby secreted into the tumor environment. The cross-talk among TMPRSS4, chemokines, and receptors will be a potential immunotherapy strategy. Our analysis revealed that TMPRSS4 is positively correlated with CCL13, CCL17, CCL18, CCL19, CCL20, CCL22, CCL23, CXCL1, CXCL2, CXCL3, CXCL5, and CX3CL1, while negatively correlated with CCL14 and CCL28. For receptors of chemokines, TMPRSS4 positively correlated with CCR1-8 and CXCR2-6 and negatively correlated with CX3CR1. Importantly, it dominantly correlated with CCR1, CCR3, CCR4, and CCR6. In principle, chemokines interact with their receptors to recruit the immune cells to the tumor location. For example, the CCL20/CCR6 interaction enhanced the invasion and migration properties of thyroid cancer cells by activating NF-κB and inducing the expression and secretion of MMP-3 [30]. CCL22, which was predominantly produced by DCs, was highly expressed in the lymph node and regulates Treg migration by binding to its receptor CCR4 [31].The results suggested that CCL22-CCR4 signaling was critical for controlling T cell immunity. CCL23 promoted angiogenesis by inducing increased CCR1 or kinase insert domain-containing receptor (KDR)/FMS-like tyrosine kinase 1 (Flk-1) on vascular endothelial cells [32, 33]. It was also associated with lymph node metastasis by inducing an increase in CCR4 expression. CCL17 and CCL22 were responsible for recruiting Treg into the tumor niche in a CCR4-dependent manner, leading to immune evasion and cancer metastasis [34]. In addition, CCL14 plays a promoting role in the activation of immune cells. Previous studies showed that it had anti- and pro-cancer dual properties. Decreased CCL14 was associated with poorer survival outcomes in HCC patients, particularly at an early stage [35]. CCL28 was a favorable prognostic factor for luminal-like cases and unfavorable for triple-negative breast cancer [36], inhibited oral squamous cell carcinoma by CCL28/RARβ [37], promoted angiogenesis in lung adenocarcinoma by CCL28/CCR3 signaling pathway [38], and even was induced to enhance the recruitment of Treg under hypoxia conditions [39]. Collectively, these findings suggest that TMPRSS4 acts as a prometastatic enzyme by modulating cross-talk between chemokines and their receptors in TC.

Despite PTC patients having an excellent clinical outcome, a small portion of patients also experience recurrence or death. Currently, cancer immune escape plays a critical role in cancer development. Cancer cells exploit immune checkpoints (CTLA-4, PD-1, PD-L1, and PD-L2) to break the physiologic maintenance of self-tolerance and inhibit the antitumor immune response. Na KJ et al. [40] found thyroid differentiation score significantly correlated with decreased immunosuppressive markers CTLA-4, PD-1, and HLA-G, and the expression of these markers was higher in BRAFV600E positive PTC patients. Immunostimulatory therapies targeting PD-1 and CTLA-4 are regarded as standard treatments for numerous malignancies with lasting antitumor effects [41]. HLA-G, which was a nonclassical major histocompatibility complex molecule with immunomodulatory properties [42], could result in immune suppression, driving tumor growth and progression by HLA-G/ILT-2/-4 (HLA-G/ILT) signaling [43]. In PTC, HLA-G expression gradually increased from hyperplasia to carcinomas. HLA-G might be a valid target for CAR-T cell therapy to specifically target and eliminate both tumor cells and HLA-G [44]. In our study, we demonstrated that TMPRSS4 positively correlated immunosuppressive markers (CTLA-4, PD-L1, HLA-G) in TC, especially for HLA-G. Our analysis may be helpful to identify promising immune therapies against TC.

Briefly, TMPRSS4 was a promising potential candidate biomarker for the immune treatment of TC in the future. Highly expressed TMPRSS4 was strongly associated with aggressive clinicopathological features, beneficial prognosis, and immune cell infiltration. Moreover, our study offers insights into a novel mechanism by which TMPRSS4 may affect the clinical outcome of TC through tumor immune infiltration but also paves the way for further studies on immunotherapy against TC. However, our current research may be limited by tumor heterogeneity, transcriptome-based cell-type quantification methods for immuno-oncology [45], and data collection methods in different database. In addition, the stratification by subtype and TC-related gene such as BRAF status was also essential for the analysis. Hence, we will further investigate when there are available independent datasets and confirm its regulatory role and mechanism in TC by performing experiments in the future.

## Supplementary Information

**Additional file 1: Supplementary Table 1.** The associations between TMPRSS4 and distinct immune populations using TIMER database. **Supplementary Table 2.** The associations between TMPRSS4 and distinct immune populations using TISIDB. **Supplementary Table 3.** The associations between TMPRSS4 and distinct immune populations using xCell. **Supplementary Table 4.** The associations between TMPRSS4 and distinct immune populations using CIBERSORT. **Supplementary Figure 1.** The correlation between TMPRSS4 and abundance of TILs in TC from TISIDB. **Supplementary Figure 2.** The correlation between TMPRSS4 and chemokines CXCL1, CXCL2, CXCL3, CXCL5, CCL14, CX3CL1, CCL19, and CCL28 in TC from TISIDB. **Supplementary Figure 3.** The correlation between TMPRSS4 and receptors of chemokines CCR2, CCR5, CCR7, CCR8, CXCR2, CXCR3, CXCR4, CXCR5, CXCR6, and CX3CR1 in TC from TISIDB. **Supplementary Figure 4**. The correlation diagram between TMPRSS4 and expression of immunosuppressive markers in TC by TIMER2.0. A: the correlation analysis without any adjustment, B: the correlation analysis adjusted by tumor purity.

## Data Availability

Publicly available datasets listed in this article can be found in the corresponding online web portal, oncomine(https://www.oncomine.org/resource/main.html), TIMER2.0(http://timer.cistrome.org/), Kaplan–Meier Plotter(http://kmplot.com/analysis), TISIDB(http://cis.hku.hk/TISIDB/index.php), UALCAN(http://ualcan.path.uab.edu/index.html).
