# Supplementary material for: TMPRSS4 is a novel biomarker and correlated with immune infiltration in thyroid carcinoma
Source: BMC Endocr Disord. 2022 Nov 16;22:280. doi: 10.1186/s12902-022-01203-3 (PMC9667668; doi:10.1186/s12902-022-01203-3)
Supplement: Supplementary file 1 — Additional file 1: Supplementary Table 1. The associations between TMPRSS4 and distinct immune populations using TIMER database. Supplementary Table 2. The associations between TMPRSS4 and distinct immune populations using TISIDB. Supplementary Table 3. The associations between TMPRSS4 and distinct immune populations using xCell. Supplementary Table 4. The associations between TMPRSS4 and distinct immune populations using CIBERSORT. Supplementary Figure 1. The correlation between TMPRSS4 and abundance of TILs in TC from TISIDB. Supplementary Figure 2. The correlation between TMPRSS4 and chemokines CXCL1, CXCL2, CXCL3, CXCL5, CCL14, CX3CL1, CCL19, and CCL28 in TC from TISIDB. Supplementary Figure 3. The correlation between TMPRSS4 and receptors of chemokines CCR2, CCR5, CCR7, CCR8, CXCR2, CXCR3, CXCR4, CXCR5, CXCR6, and CX3CR1 in TC from TISIDB. Supplementary Figure 4. The correlation diagram between TMPRSS4 and expression of immunosuppressive markers in TC by TIMER2.0. A: the correlation analysis without any adjustment, B: the correlation analysis adjusted by tumor purity. [file 12902_2022_1203_MOESM1_ESM.zip › Supplementary materials revised 20221018/Supplementary information regarding Figure1A.docx]

Figure 1A legend

Cell color was determined by the best gene rank percentile for the analysis within the cell. Saturated color block represented Top 1%, medium saturation color block represented Top 5%，and white color block represented Top 10%. We have revised the Figure1 A by adding the legend concerning color block. Note: red color represented mRNA expression level of TMPRSS4 was significantly higher in some type of cancer than its normal control, and blue color represented the significantly low expression of TMPRSS4 mRNA in some cancer. Importantly, an analysis was counted in more than one cancer type. The number 1, 2, 3 in cell represented the number of datasets in which the mRNA expression levels of TMPRSS4 were significantly up-regulated/down-regulated in patients with some type of cancer. Figure 1A included 185 analyses regarding mRNA expression detection of TMPRSS4 in cancer by Oncomine database. Specifically, 1 analysis showed evidently down-regulated TMPRSS4 in brain and CNS cancer, and 9 analyses revealed the significant up-regulated TMPRSS4 consisting of 2 analyses of Gastric cancer, 1 analysis of breast cancer, 1 analysis of cervical cancer, 1 analysis of colorectal cancer, 1 analysis of lung cancer, 3 analyses of ovarian cancer.
