# Supplementary figures and images for: TMPRSS4 is a novel biomarker and correlated with immune infiltration in thyroid carcinoma
Source: BMC Endocr Disord. 2022 Nov 16;22:280. doi: 10.1186/s12902-022-01203-3 (PMC9667668; doi:10.1186/s12902-022-01203-3)

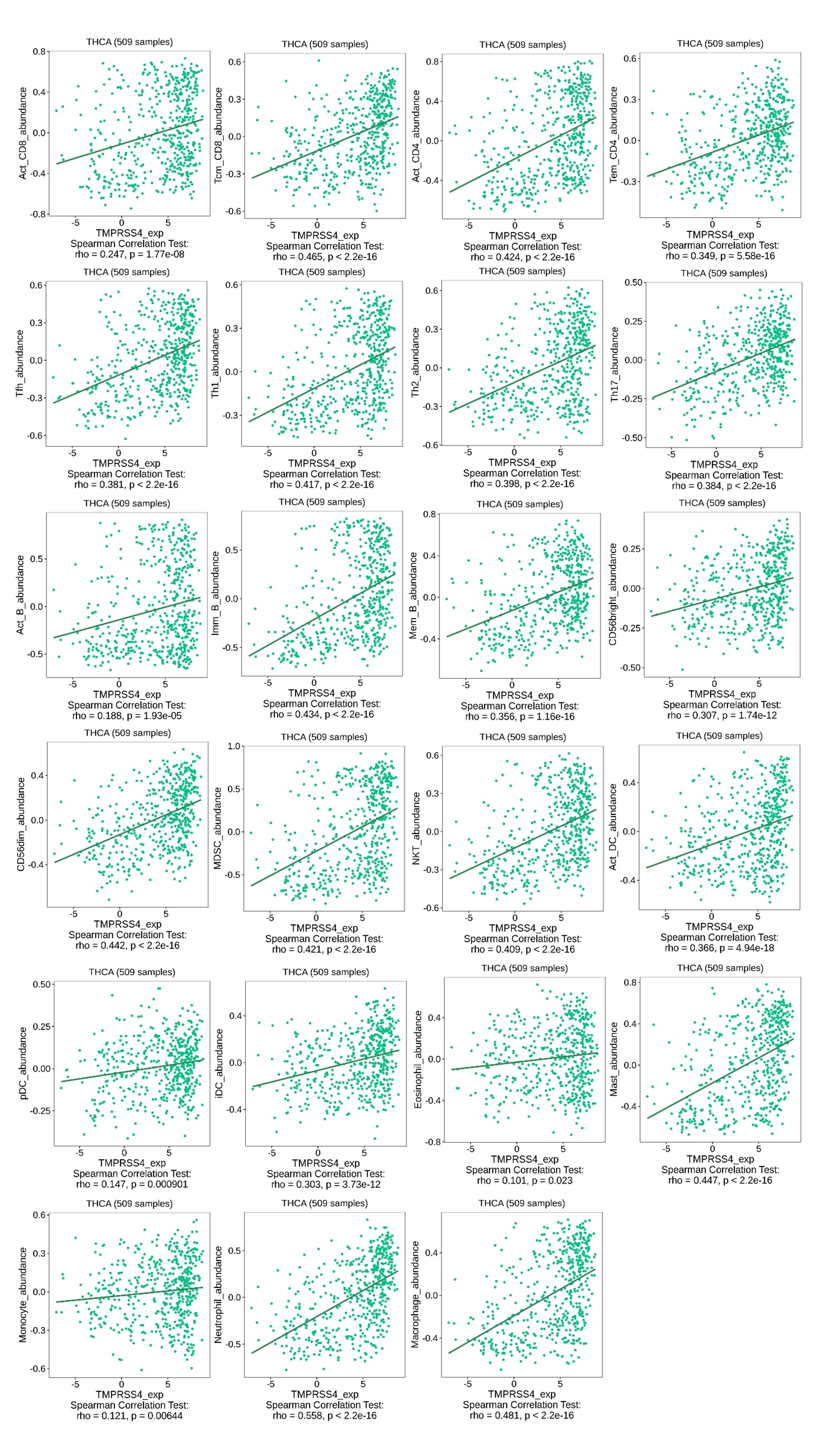

Supplement: Supplementary file 1 — Additional file 1: Supplementary Table 1. The associations between TMPRSS4 and distinct immune populations using TIMER database. Supplementary Table 2. The associations between TMPRSS4 and distinct immune populations using TISIDB. Supplementary Table 3. The associations between TMPRSS4 and distinct immune populations using xCell. Supplementary Table 4. The associations between TMPRSS4 and distinct immune populations using CIBERSORT. Supplementary Figure 1. The correlation between TMPRSS4 and abundance of TILs in TC from TISIDB. Supplementary Figure 2. The correlation between TMPRSS4 and chemokines CXCL1, CXCL2, CXCL3, CXCL5, CCL14, CX3CL1, CCL19, and CCL28 in TC from TISIDB. Supplementary Figure 3. The correlation between TMPRSS4 and receptors of chemokines CCR2, CCR5, CCR7, CCR8, CXCR2, CXCR3, CXCR4, CXCR5, CXCR6, and CX3CR1 in TC from TISIDB. Supplementary Figure 4. The correlation diagram between TMPRSS4 and expression of immunosuppressive markers in TC by TIMER2.0. A: the correlation analysis without any adjustment, B: the correlation analysis adjusted by tumor purity. [file 12902_2022_1203_MOESM1_ESM.zip › Supplementary materials revised 20221018/supplementary figure 1.tif]

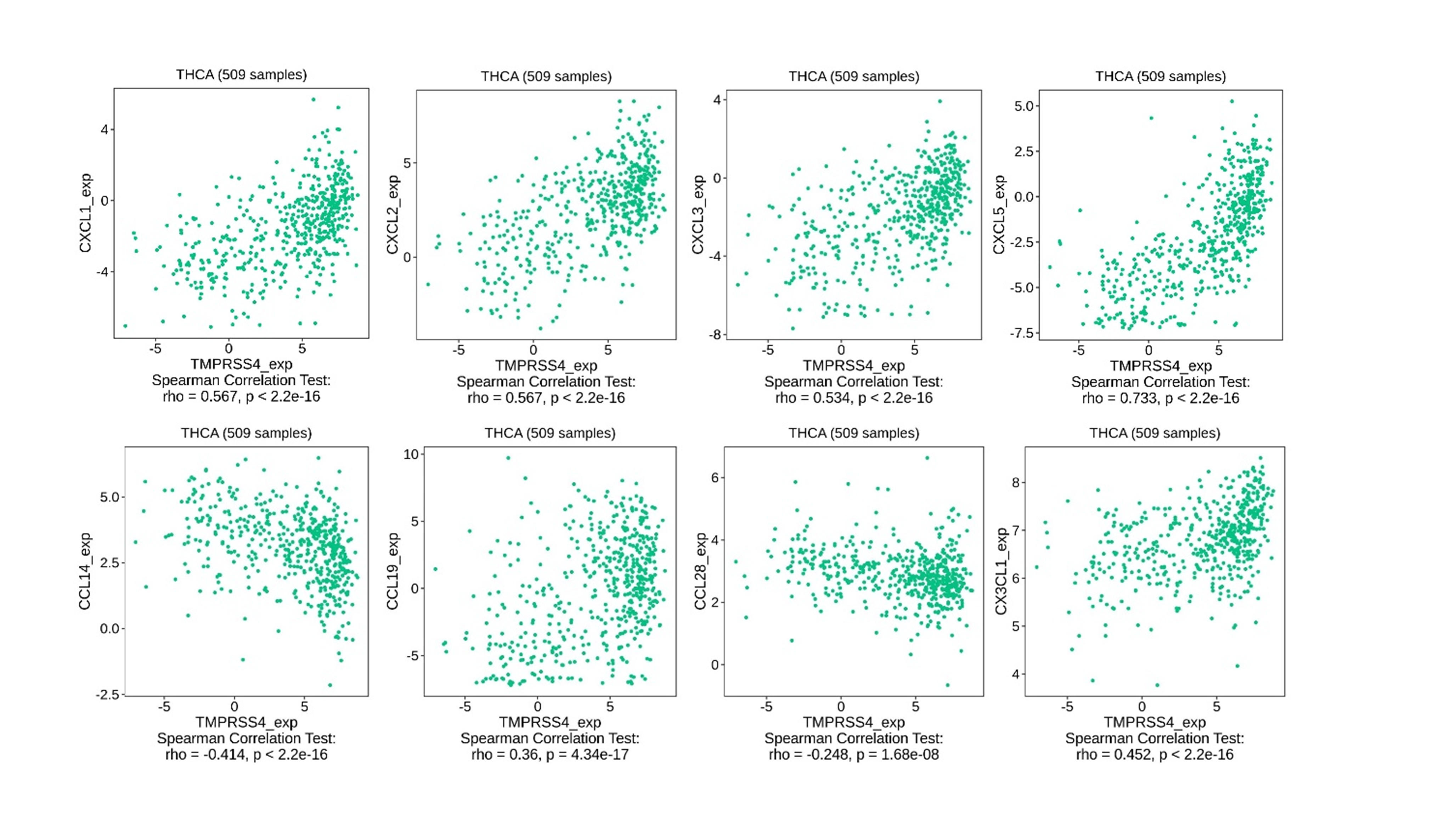

Supplement: Supplementary file 1 — Additional file 1: Supplementary Table 1. The associations between TMPRSS4 and distinct immune populations using TIMER database. Supplementary Table 2. The associations between TMPRSS4 and distinct immune populations using TISIDB. Supplementary Table 3. The associations between TMPRSS4 and distinct immune populations using xCell. Supplementary Table 4. The associations between TMPRSS4 and distinct immune populations using CIBERSORT. Supplementary Figure 1. The correlation between TMPRSS4 and abundance of TILs in TC from TISIDB. Supplementary Figure 2. The correlation between TMPRSS4 and chemokines CXCL1, CXCL2, CXCL3, CXCL5, CCL14, CX3CL1, CCL19, and CCL28 in TC from TISIDB. Supplementary Figure 3. The correlation between TMPRSS4 and receptors of chemokines CCR2, CCR5, CCR7, CCR8, CXCR2, CXCR3, CXCR4, CXCR5, CXCR6, and CX3CR1 in TC from TISIDB. Supplementary Figure 4. The correlation diagram between TMPRSS4 and expression of immunosuppressive markers in TC by TIMER2.0. A: the correlation analysis without any adjustment, B: the correlation analysis adjusted by tumor purity. [file 12902_2022_1203_MOESM1_ESM.zip › Supplementary materials revised 20221018/supplementary figure 2.tif]

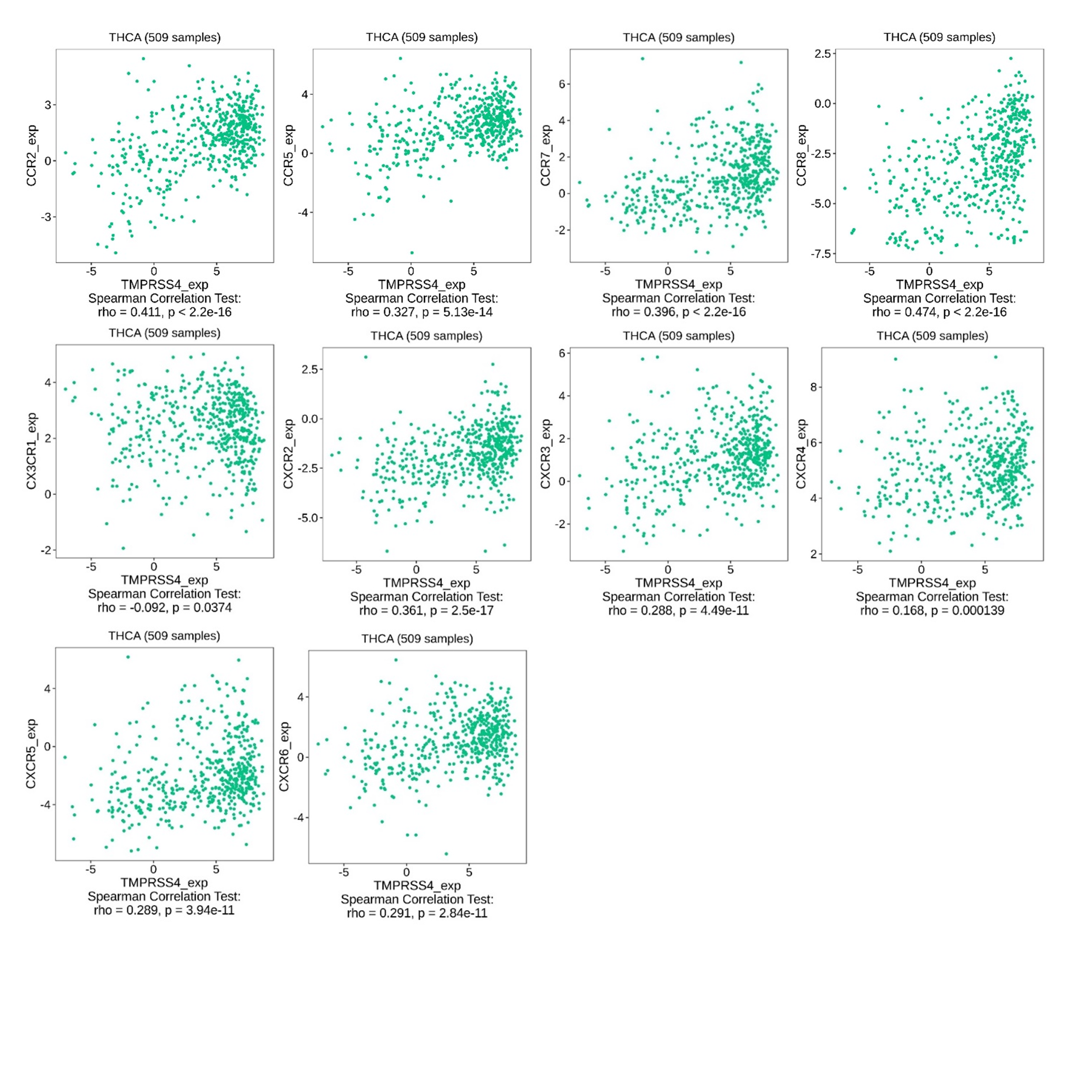

Supplement: Supplementary file 1 — Additional file 1: Supplementary Table 1. The associations between TMPRSS4 and distinct immune populations using TIMER database. Supplementary Table 2. The associations between TMPRSS4 and distinct immune populations using TISIDB. Supplementary Table 3. The associations between TMPRSS4 and distinct immune populations using xCell. Supplementary Table 4. The associations between TMPRSS4 and distinct immune populations using CIBERSORT. Supplementary Figure 1. The correlation between TMPRSS4 and abundance of TILs in TC from TISIDB. Supplementary Figure 2. The correlation between TMPRSS4 and chemokines CXCL1, CXCL2, CXCL3, CXCL5, CCL14, CX3CL1, CCL19, and CCL28 in TC from TISIDB. Supplementary Figure 3. The correlation between TMPRSS4 and receptors of chemokines CCR2, CCR5, CCR7, CCR8, CXCR2, CXCR3, CXCR4, CXCR5, CXCR6, and CX3CR1 in TC from TISIDB. Supplementary Figure 4. The correlation diagram between TMPRSS4 and expression of immunosuppressive markers in TC by TIMER2.0. A: the correlation analysis without any adjustment, B: the correlation analysis adjusted by tumor purity. [file 12902_2022_1203_MOESM1_ESM.zip › Supplementary materials revised 20221018/supplementary figure 3.tif]

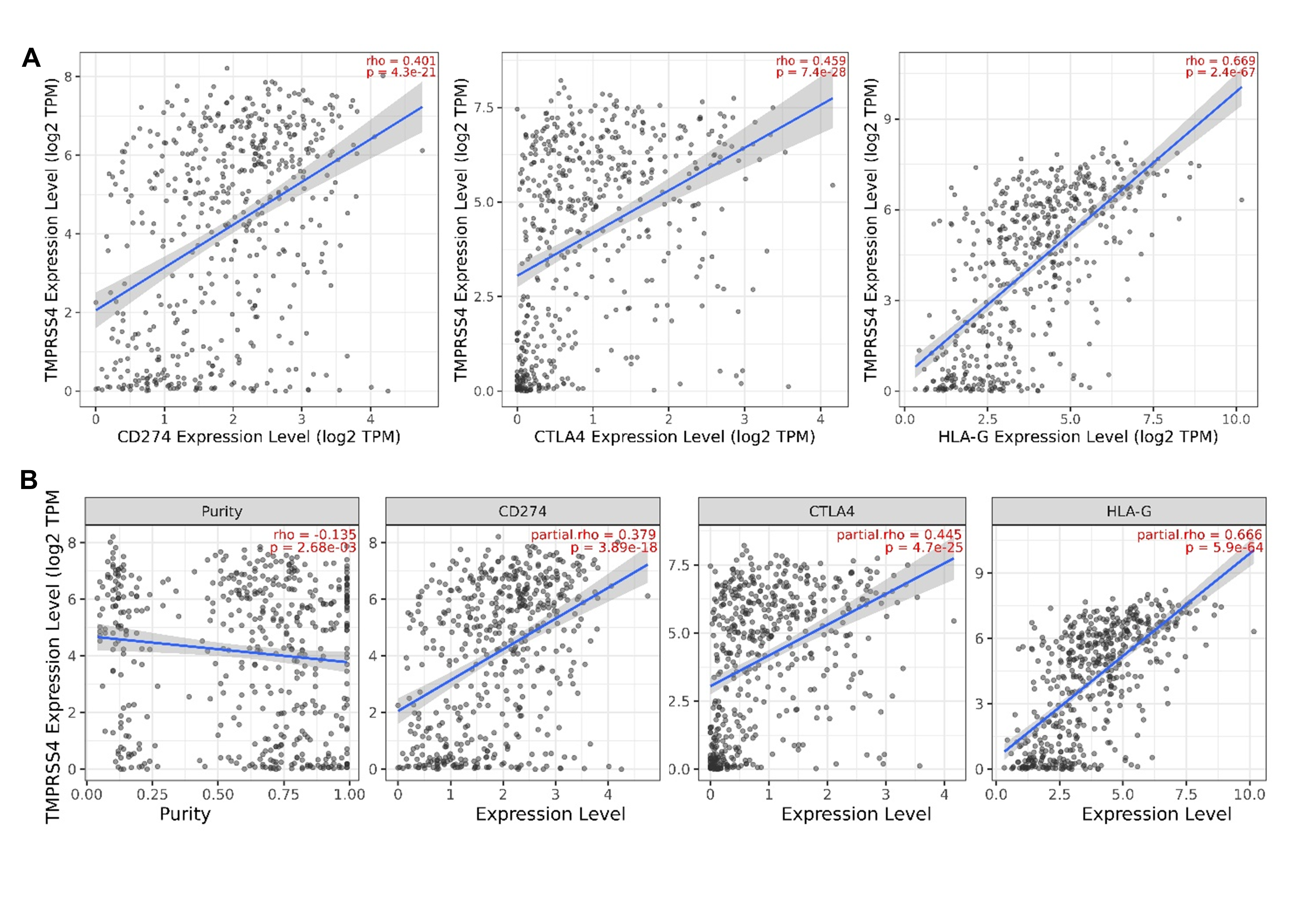

Supplement: Supplementary file 1 — Additional file 1: Supplementary Table 1. The associations between TMPRSS4 and distinct immune populations using TIMER database. Supplementary Table 2. The associations between TMPRSS4 and distinct immune populations using TISIDB. Supplementary Table 3. The associations between TMPRSS4 and distinct immune populations using xCell. Supplementary Table 4. The associations between TMPRSS4 and distinct immune populations using CIBERSORT. Supplementary Figure 1. The correlation between TMPRSS4 and abundance of TILs in TC from TISIDB. Supplementary Figure 2. The correlation between TMPRSS4 and chemokines CXCL1, CXCL2, CXCL3, CXCL5, CCL14, CX3CL1, CCL19, and CCL28 in TC from TISIDB. Supplementary Figure 3. The correlation between TMPRSS4 and receptors of chemokines CCR2, CCR5, CCR7, CCR8, CXCR2, CXCR3, CXCR4, CXCR5, CXCR6, and CX3CR1 in TC from TISIDB. Supplementary Figure 4. The correlation diagram between TMPRSS4 and expression of immunosuppressive markers in TC by TIMER2.0. A: the correlation analysis without any adjustment, B: the correlation analysis adjusted by tumor purity. [file 12902_2022_1203_MOESM1_ESM.zip › Supplementary materials revised 20221018/supplementary figure 4.tif]
