# Supplementary material for: TMPRSS4 is a novel biomarker and correlated with immune infiltration in thyroid carcinoma
Source: BMC Endocr Disord. 2022 Nov 16;22:280. doi: 10.1186/s12902-022-01203-3 (PMC9667668; doi:10.1186/s12902-022-01203-3)
Supplement: Supplementary file 1 — Additional file 1: Supplementary Table 1. The associations between TMPRSS4 and distinct immune populations using TIMER database. Supplementary Table 2. The associations between TMPRSS4 and distinct immune populations using TISIDB. Supplementary Table 3. The associations between TMPRSS4 and distinct immune populations using xCell. Supplementary Table 4. The associations between TMPRSS4 and distinct immune populations using CIBERSORT. Supplementary Figure 1. The correlation between TMPRSS4 and abundance of TILs in TC from TISIDB. Supplementary Figure 2. The correlation between TMPRSS4 and chemokines CXCL1, CXCL2, CXCL3, CXCL5, CCL14, CX3CL1, CCL19, and CCL28 in TC from TISIDB. Supplementary Figure 3. The correlation between TMPRSS4 and receptors of chemokines CCR2, CCR5, CCR7, CCR8, CXCR2, CXCR3, CXCR4, CXCR5, CXCR6, and CX3CR1 in TC from TISIDB. Supplementary Figure 4. The correlation diagram between TMPRSS4 and expression of immunosuppressive markers in TC by TIMER2.0. A: the correlation analysis without any adjustment, B: the correlation analysis adjusted by tumor purity. [file 12902_2022_1203_MOESM1_ESM.zip › Supplementary materials revised 20221018/Supplementary Table3.doc]

S**upplementary Table 3 The associations between TMPRSS4 and distinct immune**

**populations using xCell**

| **Infiltrating immune cells** | ***r*** | ***P*** |
| --- | --- | --- |
| T cell CD8+ central memory | 0.062345 | 0.169113 |
| T cell CD8+ effector memory | -0.00334 | 0.941318 |
| T cell CD8+ naive | -0.26723 | 2.00E-09*** |
| T cell CD8+ | -0.08719 | 0.05425 |
| T cell CD4+ (non-regulatory) | -0.00843 | 0.852683 |
| T cell CD4+ central memory | -0.27539 | 6.09E-10*** |
| T cell CD4+ effector memory | 0.171219 | 0.000144*** |
| T cell CD4+ memory | 0.221196 | 8.01E-07*** |
| T cell CD4+ naive | 0.012334 | 0.785797 |
| T cell CD4+ Th1 | -0.09683 | 0.032472* |
| T cell CD4+ Th2 | 0.166455 | 0.000221** |
| T cell regulatory (Tregs) | 0.313297 | 1.42E-12*** |
| B cell memory | 0.091301 | 0.043805* |
| B cell naive | -0.04564 | 0.31429 |
| B cell plasma | -0.3053 | 5.49E-12*** |
| B cell | 0.265411 | 2.60E-09*** |
| Class-switched memory B cell | 0.421538 | 1.92E-22*** |
| Neutrophil | -0.09322 | 0.039549* |
| Monocyte | 0.419969 | 2.84E-22*** |
| Macrophage M1 | 0.079351 | 0.079913 |
| Macrophage M2 | -0.21653 | 1.38E-06*** |
| Macrophage | 0.239747 | 8.28E-08*** |
| Myeloid dendritic cell activated | 0.450049 | 1.04E-25*** |
| Myeloid dendritic cell | 0.621607 | 1.66E-53*** |
| Plasmacytoid dendritic cell | -0.07899 | 0.081298 |
| NK cell | -0.01909 | 0.673994 |
| Mast cell | 0.301029 | 1.11E-11*** |
| Cancer associated fibroblast | -0.13375 | 0.003073** |
| Common lymphoid progenitor | 0.183096 | 4.73E-05*** |
| Common myeloid progenitor | -0.01458 | 0.748081 |
| Endothelial cell | -0.56353 | 3.04E-42*** |
| Eosinophil | 0.049663 | 0.273534 |
| Granulocyte-monocyte progenitor | -0.01838 | 0.68551 |
| Hematopoietic stem cell | -0.39395 | 1.45E-19*** |
| T cell gamma delta | 0.117379 | 0.00945** |
| T cell NK | 0.341271 | 8.93E-15*** |

*: *P* < 0.05, **: *P* < 0.01, ***: *P* < 0.001
